# Evaluation of T Cell Receptor Construction Methods from scRNA-Seq Data

**DOI:** 10.1093/gpbjnl/qzae086

**Published:** 2024-12-12

**Authors:** Ruonan Tian, Zhejian Yu, Ziwei Xue, Jiaxin Wu, Lize Wu, Shuo Cai, Bing Gao, Bing He, Yu Zhao, Jianhua Yao, Linrong Lu, Wanlu Liu

**Affiliations:** Department of Rheumatology and Immunology of the Second Affiliated Hospital, and Centre of Biomedical Systems and Informatics of Zhejiang University-University of Edinburgh Institute, Zhejiang University School of Medicine, Hangzhou 310003, China; Future Health Laboratory, Innovation Center of Yangtze River Delta, Zhejiang University, Jiaxing 314100, China; Department of Rheumatology and Immunology of the Second Affiliated Hospital, and Centre of Biomedical Systems and Informatics of Zhejiang University-University of Edinburgh Institute, Zhejiang University School of Medicine, Hangzhou 310003, China; Department of Rheumatology and Immunology of the Second Affiliated Hospital, and Centre of Biomedical Systems and Informatics of Zhejiang University-University of Edinburgh Institute, Zhejiang University School of Medicine, Hangzhou 310003, China; Future Health Laboratory, Innovation Center of Yangtze River Delta, Zhejiang University, Jiaxing 314100, China; Department of Rheumatology and Immunology of the Second Affiliated Hospital, and Centre of Biomedical Systems and Informatics of Zhejiang University-University of Edinburgh Institute, Zhejiang University School of Medicine, Hangzhou 310003, China; Future Health Laboratory, Innovation Center of Yangtze River Delta, Zhejiang University, Jiaxing 314100, China; Institute of Immunology and Department of Dermatology and Rheumatology at Sir Run Run Shaw Hospital, Zhejiang University School of Medicine, Hangzhou 310058, China; Department of Rheumatology and Immunology of the Second Affiliated Hospital, and Centre of Biomedical Systems and Informatics of Zhejiang University-University of Edinburgh Institute, Zhejiang University School of Medicine, Hangzhou 310003, China; Department of Rheumatology and Immunology of the Second Affiliated Hospital, and Centre of Biomedical Systems and Informatics of Zhejiang University-University of Edinburgh Institute, Zhejiang University School of Medicine, Hangzhou 310003, China; AI Lab, Tencent, Shenzhen 518000, China; AI Lab, Tencent, Shenzhen 518000, China; AI Lab, Tencent, Shenzhen 518000, China; Future Health Laboratory, Innovation Center of Yangtze River Delta, Zhejiang University, Jiaxing 314100, China; Institute of Immunology and Department of Dermatology and Rheumatology at Sir Run Run Shaw Hospital, Zhejiang University School of Medicine, Hangzhou 310058, China; Shanghai Immune Therapy Institute, Shanghai Jiao Tong University School of Medicine Affiliated Renji Hospital, Shanghai 200025, China; Department of Rheumatology and Immunology of the Second Affiliated Hospital, and Centre of Biomedical Systems and Informatics of Zhejiang University-University of Edinburgh Institute, Zhejiang University School of Medicine, Hangzhou 310003, China; Future Health Laboratory, Innovation Center of Yangtze River Delta, Zhejiang University, Jiaxing 314100, China

**Keywords:** T cell receptor, scRNA-seq, Benchmark analysis, TCR construction, Adaptive immunity

## Abstract

T cell receptors (TCRs) serve key roles in the adaptive immune system by enabling recognition and response to pathogens and irregular cells. Various methods have been developed for TCR construction from single-cell RNA sequencing (scRNA-seq) datasets, each with its unique characteristics. Yet, a comprehensive evaluation of their relative performance under different conditions remains elusive. In this study, we conducted a benchmark analysis utilizing experimental single-cell immune profiling datasets. Additionally, we introduced a novel simulator, YASIM-scTCR (Yet Another SIMulator for single-cell TCR), capable of generating scTCR-seq reads containing diverse TCR-derived sequences with different sequencing depths and read lengths. Our results consistently showed that TRUST4 and MiXCR outperformed others across multiple datasets, while DeRR demonstrated considerable accuracy. We also discovered that the sequencing depth inherently imposes a critical constraint on successful TCR construction from scRNA-seq data. In summary, we present a benchmark study to aid researchers in choosing the appropriate method for reconstructing TCRs from scRNA-seq data.

## Introduction

T cell receptors (TCRs) play central roles in recognizing pathogen- and self-derived antigens, thereby fostering immunosurveillance of infectious diseases, autoimmune disorders, and cancers [1,2]. TCRs are highly diverse due to V(D)J recombination and the pairing of α/β chains, especially in the antigen-recognizing complementary-determining region 3 (CDR3), leading to an enormous TCR repertoire capable of recognizing a wide range of antigens [3,4]. Therefore, characterizing the antigen receptor repertoire and understanding its dynamics in disease progression are crucial for guiding vaccine design and developing precise immunotherapy [5,6].

Next-generation sequencing (NGS)-based bulk TCR sequencing (TCR-seq) enables the characterization of antigen receptor repertoires under different disease conditions [7–9]. However, the lack of paired information for α/β chains limits its application for experimental validation. Emerging single-cell technologies offer a promising approach for capturing gene expression profiles (GEX) alongside the information of paired TCR α/β chains at the single-cell level, referred to as single-cell immune profiling (scRNA+TCR-seq) [9–11]. This approach potentially provides profound insights into antigen receptor repertoire analysis and helps reveal functional TCRs [9,10]. While single-cell immune profiling data based on 10X and SMART-seq methodologies have seen exponential growth, its widespread application is still restrained due to the high cost and additional TCR amplification steps [9,12,13]. For instance, in our human Antigen Receptor database (huARdb) [14], we only collect around half a million T cells, significantly fewer than the theoretically predicted TCR diversity of 1 × 10^20^ [15]. Meanwhile, scRNA-seq datasets of T cells, inherently containing TCR sequence information, are abundantly available in public databases [11,16]. Nevertheless, TCR-derived sequences are frequently disregarded in most scRNA-seq studies as they might not constitute the primary focus of investigations. However, it is worth noting that even with lower capture efficiency, scRNA-seq may still offer researchers the potential to reconstruct TCR sequence information from the scRNA-seq data, which could significantly enhance our understanding for T cell function [17,18].

TCR construction methods share certain similarities with *de novo* transcriptome assembly and contig annotation, such as Trinity [12] and IgBLAST [19]. However, it also diverges from these general-purpose methods. For instance, there is a lack of reliable reference for the highly variable CDR3 regions [3]. Furthermore, a specific reference tailored to TCR sequences is essential for accurate analysis [10]. These distinct characteristics render TCR assembly and annotation more challenging, highlighting the need for specialized methods [10].

Most methods construct TCRs through candidate read identification, TCR read assembly, and V(D)J gene annotation. To facilitate a comprehensive summary of method characteristics, we conducted a thorough review of all available methods capable of reconstructing TCRs and B cell receptors (BCRs) from bulk and single-cell RNA sequencing [(sc)RNA-seq] datasets, including MiXCR [20], TraCeR [21], VDJer [22], BASIC [23], BALDR [24], BraCeR [25], VDJPuzzle [26], ImRep [27], CATT [28], TRUST4 [29], and DeRR (Table S1). While BALDR, BraCeR, and VDJer exclusively support BCR construction, TraCeR and DeRR are specifically designed for TCR construction. Generally, these methods can be categorized based on whether they implement a *de novo* algorithm while constructing TCRs/BCRs. For example, MiXCR, VDJer, ImRep, CATT, and TRUST4 adopt a *de novo* approach for read alignment, assembly, and annotation. Other methods, such as TraCeR, BASIC, BLADR, BraCeR, VDJPuzzle, and DeRR, construct a pipeline by incorporating existing methods for such task. Commonly used methods for read alignment in such pipelines include Bowtie [30], Bowtie2 [31], BWA [32], and STAR [33], with Trinity [12] for assembly, and IgBLAST [19] for V/J gene and CDR3 amino acid sequence annotation.

To provide resources for researchers in choosing suitable TCR construction methods, we conducted extensive evaluation of the performance of current TCR construction methods on scRNA-seq, scTCR-seq, pseudo-bulk RNA-seq, bulk TCR-seq, and simulated scTCR-seq data. The seven methods included in this benchmark study were MiXCR, TraCeR, BASIC, ImRep, CATT, TRUST4, and DeRR. Leveraging previously published scRNA+TCR-seq datasets, we utilized scRNA-seq data as input for various methods and employed scTCR-seq dataset as ground truth to assess their accuracy and sensitivity, focusing on CDR3 amino acid sequences and V/J gene usages. Additionally, we generated pseudo-bulk RNA-seq data to examine the effect of cell number on TCR construction accuracy. Furthermore, we developed a simulator called YASIM-scTCR (Yet Another SIMulator for single-cell TCR) to generate scTCR-seq reads containing TCR- and non-TCR-derived sequences, thus allowing us to evaluate method performance under various sequencing depths and read lengths. Finally, we also assessed the computational efficiency of different methods. In conclusion, our analyses established the quality control metrics for the precise assembly of TCRs from scRNA-seq data.

## Results

### Overview of methods supporting TCR reconstruction from scRNA-seq data

**Figure 1:**
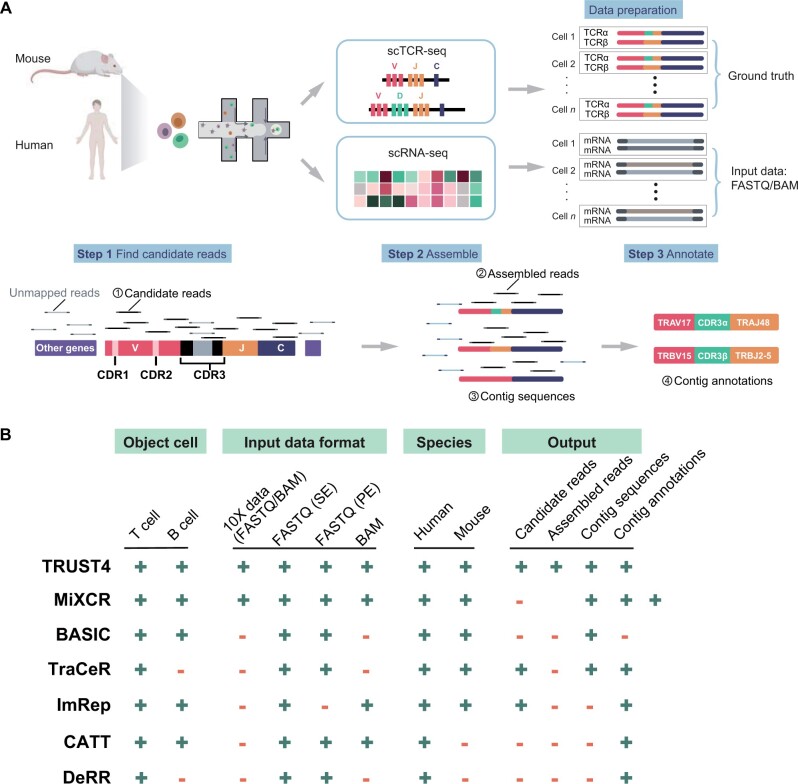
A benchmark framework for TCR construction methods using real scRNA-seq data **A**. Schematic representation of the benchmark workflow. Human and mouse scRNA-seq and scTCR-seq datasets were used as input data and ground truth, respectively. The analytical pipeline comprises three key steps: Step 1, find candidate reads from the input data; Step 2, assemble contigs from all candidate reads with partial overlaps; Step 3, annotate the contig sequences and reconstruct TCRs. **B**. Comprehensive evaluation of the adaptability of various methods with a focus on four key aspects: object cell, input data format, species, and output. “+” represents the presence of this feature, and “-” represents the absence of this feature. CDR, complementary-determining region; BAM, Binary Alignment Map format; TCR, T cell receptor; scRNA-seq, single-cell RNA sequencing; scTCR-seq, single-cell TCR sequencing; SE, single-end sequencing; PE, paired-end sequencing.

To evaluate the performance of various TCR construction methods, we compiled previously published single-cell immune profiling datasets containing paired scRNA-seq and scTCR-seq data from both human and mouse (Table S2). By subjecting 10X scRNA-seq data to different TCR construction methods and comparing the assembled TCR with its paired scTCR-seq data, we thoroughly assessed the accuracy and sensitivity of each method (**Figure 1**A). In general, the TCR construction algorithms can be categorized into three main steps. The first step involves searching for candidate TCR-derived reads from FASTQ or BAM files using a reference for TCR segments. They are then assembled to TCR contigs using methods like de Bruijn Graph [34]. Lastly, V(D)J segments are identified and sequences of CDR3 regions are annotated (Figure 1A).

In this benchmark study, we comprehensively analyzed the performance of methods designed for TCR construction from (sc)RNA-seq data. Among these, six have been previously published in peer-reviewed literature [20,21,23,27–29], while DeRR is publicly available from https://github.com/GuoBioinfoLab/DeRR. We first summarized the adaptability of these methods across four different aspects: object cell type (T/B cell), input data format (FASTQ/BAM), species supported (human/mouse), and output files (Figure 1B). Regarding the input data format, all methods support raw FASTQ format, while a subset of them (TRUST4, MiXCR, ImRep, and CATT) also accommodate BAM format. While all methods are compatible with demultiplexed scRNA-seq data (one FASTQ/BAM per cell, *e.g.*, SMART-seq), it is worth noting that only MiXCR and TRUST4 support 10X scRNA-seq (Figure 1B). For the output files, we focused on candidate read sequences, assembled read sequences, contig sequences, and contig annotations, as they play a crucial role in assessing methods’ performance (Figure 1B). TRUST4 demonstrated a commendable capability in offering comprehensive output at each stage, while BASIC only provided the contig sequences without any annotation, leading us to exclude it in our study (Figure 1B).

### Accuracy and sensitivity of different methods using experimental scRNA-seq data

**Figure 2:**
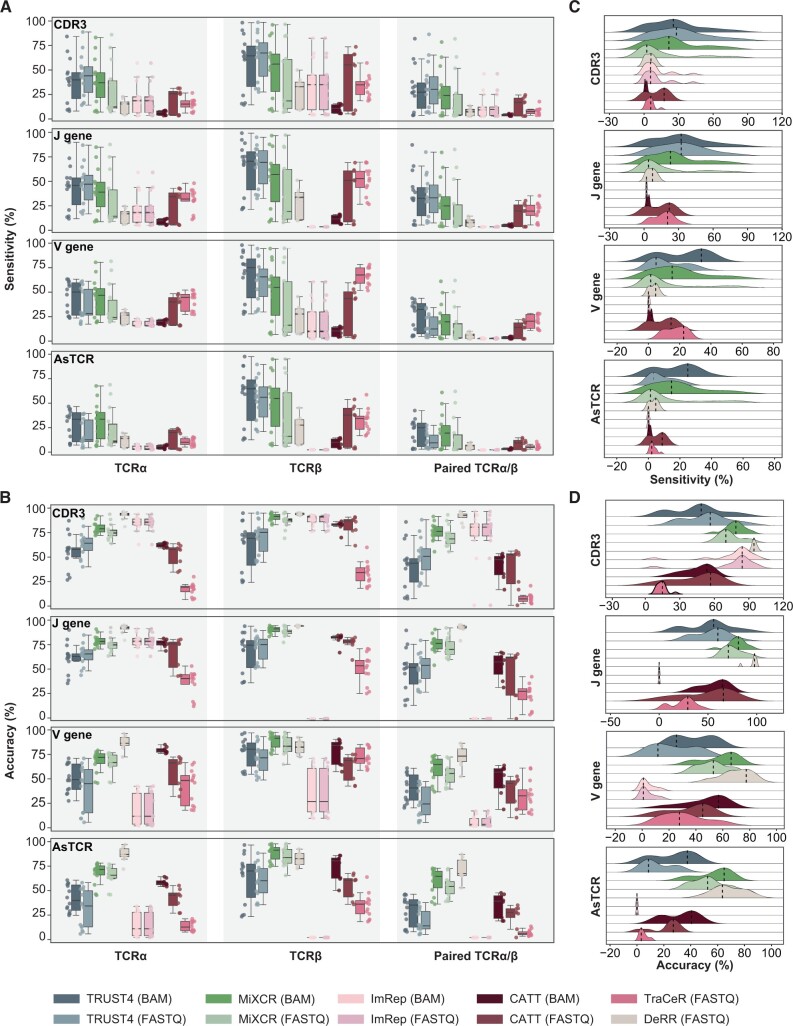
Comparative analysis of TCR construction methods on scRNA-seq data **A**. Box plot showing the sensitivity of TCR prediction of different methods. The line in the middle represents the median; the bottom and the top of the box represent the 25th and 75th percentiles, respectively; and whiskers represent the minimum and maximum points within 1.5 times the interquartile range. **B**. Box plot showing the accuracy of TCR prediction of different methods. **C**. Ridge plot showing the sensitivity distribution of CDR3, V gene, J gene, and AsTCR of paired TCRα/β. The vertical dashed line marks the peak for each method. **D**. Ridge plot showing the accuracy distribution of CDR3, V gene, J gene, and AsTCR of paired TCRα/β. AsTCR, assembled TCR.

To investigate the impact of input file format, we evaluated various methods using both FASTQ and genome-aligned BAM files from scRNA-seq data (**Figure 2**). We assessed the accuracy and sensitivity of CDR3 amino acid sequences, V/J gene calling, and assembled TCRs (AsTCRs) for both α and β chains from human and mouse (Figure 2; Table S2). The results revealed that TRUST4 (FASTQ/BAM) and MiXCR (BAM) demonstrated consistently high sensitivity for both α and β chains (Figure 2A). MiXCR (FASTQ), CATT (FASTQ), DeRR (FASTQ), and TraCeR (FASTQ) exhibited commendable performance in CDR3 assembly and V/J gene calling, while CATT (BAM) showed comparatively lower sensitivity in this context (Figure 2A). ImRep (FASTQ/BAM) also exhibited acceptable performance for CDR3 assembly. However, it only reported J genes without a specific subgroup for the β chains leading to uncharacterized TRBJ gene calling (Figure 2A). The performance of methods in CDR3 assembly and V/J gene calling was generally consistent, with DeRR and MiXCR displaying the highest accuracy on AsTCRs (Figure 2B).

For both accuracy and sensitivity evaluation, our results revealed that β chains overall outperformed α chains (Figure 2A and B). This observation may be attributed to several factors. Firstly, α chains typically display lower expression levels (Figure S1A). Furthermore, β chains often receive more attention from researchers, potentially leading to a preference in the curation of reference sequences and development of TCR construction algorithms. We also observed discrepancy in performance between FASTQ and BAM format, which may be attributed to the discarding of reads from highly variable CDR3 regions during alignment (Figure 2A and B). In summary, TRUST4 and MiXCR demonstrated notably higher sensitivity for TCR construction, while DeRR and MiXCR exhibited relatively high accuracy levels.

For non-droplet-based protocols, we used scRNA-seq datasets from 50 T cells generated by SMART-seq and performed TCR assembly (Table S2). Since paired scTCR-seq data for SMART-seq are unavailable, we instead quantified the number of cells with AsTCRs and assessed their overlap (Figure S2A–D). TRUST4 assembled the largest number of AsTCRs, and displayed high overlap with MiXCR for TRAV, TRBV, and TRBJ calling (Figure S2A–D).

We observed substantial variability in performance across different scRNA-seq datasets (Figure 2C and D). Moreover, all the evaluated methods showed limited sensitivity in TCR construction (Figure 2A). These findings prompted us to explore the underlying factors. (1) How do characteristics of scRNA-seq data, such as species origin, sequencing strategies (paired-end or single-end), sequencing length, and sequencing depth, influence the performance of these methods? (2) Is the limitation associated with the algorithms themselves, or could it be attributed to the low capture efficiency of TCR sequences in scRNA-seq data?

### Accuracy and sensitivity of different methods using simulated scTCR-seq data

**Figure 3:**
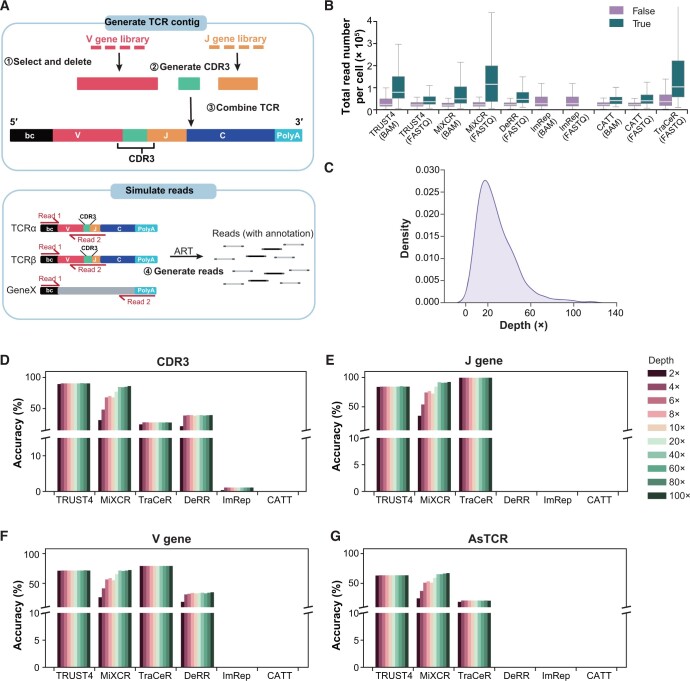
Performance comparison across real and simulated datasets with different sequencing depths **A**. Schematic representation of simulating scTCR-seq and scRNA-seq datasets. Red, green, orange, dark blue, light blue, black, and gray boxes represent V gene, CDR3 sequence, J gene, C gene, polyA sequence, cell barcode sequence, and non-TCR genes, respectively. **B**. Box plot showing the total read number of cells with successfully (True) and unsuccessfully (False) assembled TCRs on the collected experimental scRNA-seq datasets. The line in the middle represents the median; the bottom and the top of the box represent the 25th and 75th percentiles, respectively; and whiskers represent the minimum and maximum points within 1.5 times the interquartile range. **C**. Density plot showing the sequencing depth distribution of TCR sequences in TCR-assembled cells obtained from TRUST4. **D**. Bar plot showing the accuracy of paired CDR3 reconstruction on the simulated datasets with different sequencing depths. **E**. Bar plot showing the accuracy of paired J gene reconstruction on the simulated datasets with different sequencing depths. **F**. Bar plot showing the accuracy of paired V gene reconstruction on the simulated datasets with different sequencing depths. **G**. Bar plot showing the accuracy of paired AsTCR reconstruction on the simulated datasets with different sequencing depths. PolyA, a stretch of adenine-rich nucleotides added to the 3′ end of RNA molecules.

We developed a simulation framework, YASIM-scTCR, based on parameters estimated from huARdb [14,35]. Using YASIM-scTCR, we generated scTCR-seq data containing TCR-derived reads (**Figure 3**A). In this simulation framework, YASIM-scTCR initially generates TCR contigs by adhering to the principles of TCR recombination (Figure 3A). Specifically, V and J genes are selected from the human V and J gene reference annotations, and deletions are then introduced at the V gene-CDR3 (V-CDR3) and CDR3-J gene (CDR3-J) junctions. Subsequently, CDR3 sequences are generated and combined with the clipped V/J gene segments to form TCR contigs. To ensure the simulation of TCRs with realistic characteristics, we analyzed and adopted the length distribution of TCRs and CDR3 regions using publicly available single-cell immune profiling datasets [14,35] (Figure S3A–C). To control the number of deleted amino acids at the V-CDR3 and CDR3-J junctions, we evaluated the frequency distribution for the length of deleted amino acids and J genes (Figure S3D and E). Additionally, we assessed and simulated amino acid preferences in the CDR3 regions of TCRα/β (Figure S3F and G). We also evaluated and simulated the distribution of V/J gene usage bias (Figure S4). Subsequently, we constructed complete TCR contigs based on the V(D)J sequences, incorporating C gene and cell barcode information, in line with the 5′ 10X Genomics scTCR-seq library construction strategy (Figure 3A). After generating the TCR contigs, YASIM-scTCR would simulate gene expression profiles for protein-coding genes based on experimental scRNA-seq data using scDesign2 [36]. Lastly, by employing ART [37], a tool for generating synthetic NGS reads, we compiled the simulated scTCR-seq data containing TCR- and non-TCR-derived reads (Figure 3A). Moreover, YASIM-scTCR offers flexibility in the simulation process by allowing the adjustment of various parameters, such as read length and sequencing depth over TCRs.

Using YASIM-scTCR, we conducted an in-depth analysis of candidate reads and their impact on TCR construction performance. TRUST4, TraCeR, and ImRep were able to generate outputs for candidate reads. For other methods, such as DeRR, we modified the source code to enable candidate read output. Subsequently, we assessed the efficiency of candidate read identification and the final TCR construction accuracy using simulated scTCR-seq data from 500 cells. In candidate read identification, TRUST4 and TraCeR displayed exceptional performance, achieving 100% for both true positive rates (TPRs) and true negative rates (TNRs) (Figure S5A). DeRR also achieved a high TPR (100%), although its TNR was minimal (< 0.01%) (Figure S5A). Conversely, ImRep exhibited a high TNR (96%) along with a low TPR (68%), which may have affected its overall TCR construction performance (Figure S5A). On simulated data, TCR construction accuracy generally aligned with the TPR in candidate read identification (Figure S5B). Specifically, TRUST4 outperformed in CDR3 assembly and AsTCR construction for both chains, while TraCeR achieved high accuracy in V/J gene calling (Figure S5B). DeRR achieved approximately 20%–40% accuracy in AsTCR construction, whereas ImRep displayed less than 5% accuracy (Figure S5B). We also observed that all methods, particularly TRUST4, performed better on the simulated data than on experimental data. A potential explanation for this discrepancy is that YASIM-scTCR simulates 400× scTCR-seq reads, which contain more TCR-derived reads compared to the experimental scRNA-seq data. This observation partially supports our hypothesis that the low TCR construction rate in scRNA-seq data is more likely due to the low capture efficiency of TCR sequences in the data, rather than inherent deficiencies in the algorithms.

### Performance analysis of different methods across various species origins, sequencing strategies, read lengths, and depths

To investigate potential factors contributing to the varying performance of TCR construction methods, we first visualized the results from human and mouse data separately. While most TCR construction methods exhibited comparable performance for both species, notable heterogeneity within each species suggested that additional factors may influence the performance of the methods (Figure S6A and B).

To assess the impact of scRNA-seq library construction strategies, we segregated performance of the methods based on the sequencing strategies either single-end and paired-end sequencing corresponding to the 10X Single Cell 3′ and 5′ Gene Expression (GEX) library preparation protocols, respectively (Tables S3 and S4). Our comparative analysis revealed that paired-end sequencing data demonstrated higher accuracy and sensitivity compared to single-end sequencing data (Figure S6C and D). This observation aligns with the results from single-cell immune profiling techniques, suggesting that 3′ GEX library construction strategies may inadequately capture full-length TCR sequences. Consequently, we recommend the use of scRNA-seq data from the 5′ GEX library for TCR construction.

Next, we investigated the influence of read length for TCR construction using simulated scTCR-seq data with varying read lengths, while maintaining a fixed sequencing depth of 400×. The tested read lengths included 100 bp, 150 bp, and 250 bp (Figure S7). A read length of 150 bp achieved approximately 100% accuracy for TCR construction (Figure S7A and B). Notably, a 100-bp read length fell short for TCR construction in 5′ scTCR-seq libraries. In such cases, only J genes can be reliably identified due to their proximity to the 5′ end of sequencing reads (Figure 3A).

Furthermore, we explored the significance of sequencing depth on the performance of the methods. Initially, we compared the sequencing depth differences between cells with and without AsTCRs in experimental scRNA-seq data. Cells with correct AsTCRs displayed deeper sequencing depth (Figure 3B, Figure S8A). To estimate the TCR sequencing depth in experimental scRNA-seq data, we calculated TCR contig depths using TRUST4 given its outstanding performance (Figure 3C). Based on this estimation, we simulated data with varying sequencing depths, ranging from 2× to 100×. The results demonstrated that most methods exhibited higher TCR assembly accuracy with increased sequencing depth. However, we observed distinct patterns for successfully assembled ratios under varying sequencing depths (Figure 3D–G, Figure S8B and C). For instance, TRUST4 and TraCeR achieved optimal results at 2×, while MiXCR required deeper sequencing depths of approximately 100× (Figure 3D–G, Figure S8B and C). In contrast, DeRR demonstrated adequate performance in assembly results at 400× (Figure S7A and B). For CATT and ImRep, which showed inferior assembly outcomes for experimental scRNA-seq data (Figure 2), their performance further declined when applied to simulated scTCR-seq data, likely indicating unsuitability for scTCR-seq data. These findings underscore the significance of sequencing depth for TCR construction and provide a filtering cut-off criterion. Specifically, for TRUST4, scRNA-seq data with a total read count exceeding 100,000 per cell or TCR sequencing depth greater than 2× are more likely to result in successful TCR assembly.

### Performance analysis of different methods using (sc)TCR-seq data

The performance of most methods was notably superior on simulated data compared to experimental data. This discrepancy can largely be attributed to the limited presence of TCR-related reads in the experimental data. Due to TCR-specific amplification, (sc)TCR-seq data typically contains adequate TCR-related reads. Therefore, we also assessed the TCR assembly performance on (sc)TCR-seq data (Table S2).

**Figure 4:**
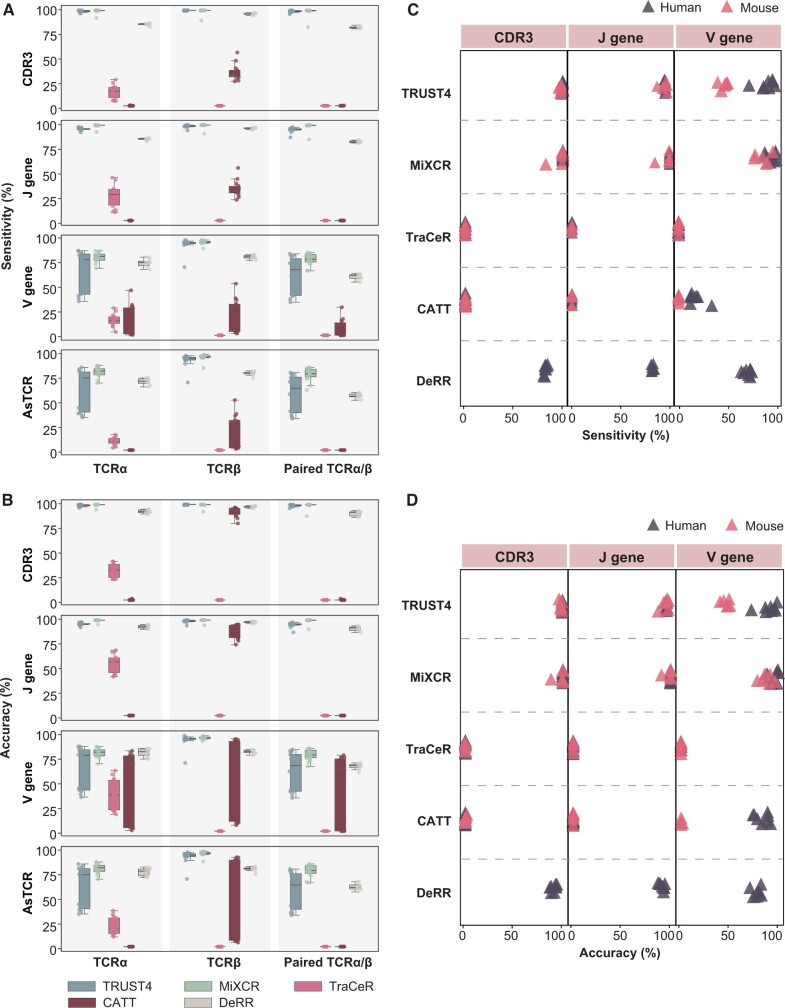
Comparative analysis of TCR construction methods on scTCR-seq data **A**. Box plot showing the sensitivity of TCR prediction of different methods. The line in the middle represents the median; the bottom and the top of the box represent the 25th and 75th percentiles, respectively; and whiskers represent the minimum and maximum points within 1.5 times the interquartile range. **B**. Box plot showing the accuracy of TCR prediction of different methods. **C**. Dot plot represent the sensitivity of different methods for mouse and human data. **D**. Dot plot represent the accuracy of different methods for mouse and human data.

For scTCR-seq data, MiXCR displayed sensitivity and accuracy close to 100%, while the results of TRUST4 and DeRR were slightly inferior (**Figure 4**A–D). In the assessment of bulk TCR-seq data, TRUST4 and MiXCR assembled the highest number of CDR3β and also showed the most overlap between them (Figure S9A–D).

In summary, TRUST4 and MiXCR demonstrated commendable performance in TCR assembly for both scTCR-seq and bulk TCR-seq data. They proved to be suitable not only for scRNA-seq data with limited TCR-related reads but also for TCR-seq data with abundant TCR-related reads.

### Performance analysis of different methods using pseudo-bulk RNA-seq data with varying cell numbers

**Figure 5:**
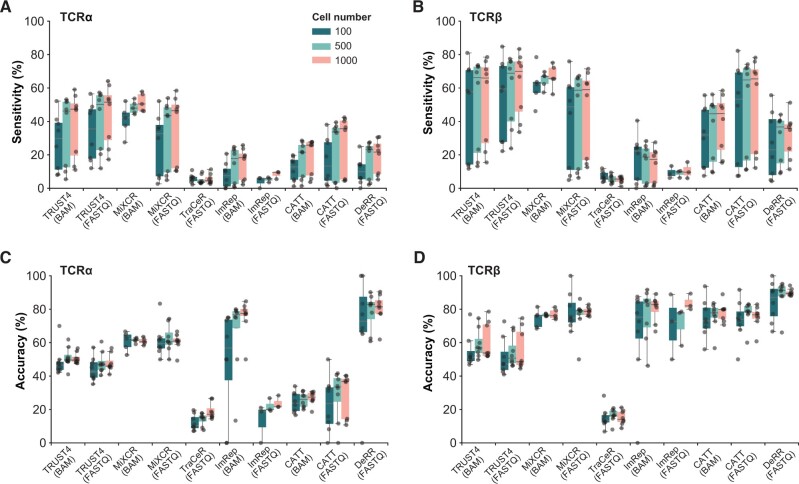
Performance assessment of different methods on pseudo-bulk RNA-seq data with varying cell numbers **A**. Box plot showing the sensitivity of TCRα assembly by different methods at varying cell numbers. The line in the middle represents the median; the bottom and the top of the box represent the 25th and 75th percentiles, respectively; and whiskers represent the minimum and maximum points within 1.5 times the interquartile range. **B**. Box plot showing the sensitivity of TCRβ assembly by different methods at varying cell numbers. **C**. Box plot showing the accuracy of TCRα assembly by different methods at varying cell numbers. **D**. Box plot showing the accuracy of TCRβ assembly by different methods at varying cell numbers.

In light of the substantial amount of available bulk RNA-seq data and the crucial role of bulk T-cell repertoire analysis in diverse diseases [38–40], it is therefore essential to evaluate the performance of these TCR construction methods on bulk RNA-seq data. Consequently, we generated pseudo-bulk RNA-seq data derived from experimental scRNA-seq data to assess the performance of these methods across varying cell numbers, including 100, 500, and 1000 cells. TRUST4 exhibited exceptional sensitivity in both scRNA-seq and pseudo-bulk RNA-seq data. MiXCR demonstrated higher sensitivity and accuracy specifically for pseudo-bulk RNA-seq data in contrast to scRNA-seq data. CATT displayed notable sensitivity, particularly in the TCRβ assembly, in pseudo-bulk RNA-seq data. Furthermore, DeRR showcased highest accuracy in the identification of both CDR3α and CDR3β in pseudo-bulk RNA-seq data, while its sensitivity remained comparatively modest concerning other methods (**Figure 5**). Generally, our findings revealed that most methods displayed increased accuracy and sensitivity as the cell number increased (Figure 5). Moreover, similar to the observations in scRNA-seq data, β chains tend to exhibit higher accuracy than α chains in TCRs assembled from pseudo-bulk RNA-seq data (Figure 5).

TCR abundance may be a crucial factor influencing the correct assembly of TCRs. Therefore, we evaluated the sensitivity and accuracy of pseudo-bulk RNA-seq data with varying TCR abundance (Figure S10). We categorized cells in each sample into three equal-sized groups (high, medium, and low) based on their TCR abundance, and compared the sensitivity and accuracy among different groups. The results indicated that higher TCR abundance was associated with increased performance in most cases. Additionally, TRUST4 and MiXCR consistently performed relatively well across different TCR abundance levels compared to other methods.

### Overall scoring and ranking of TCR construction methods

**Figure 6:**
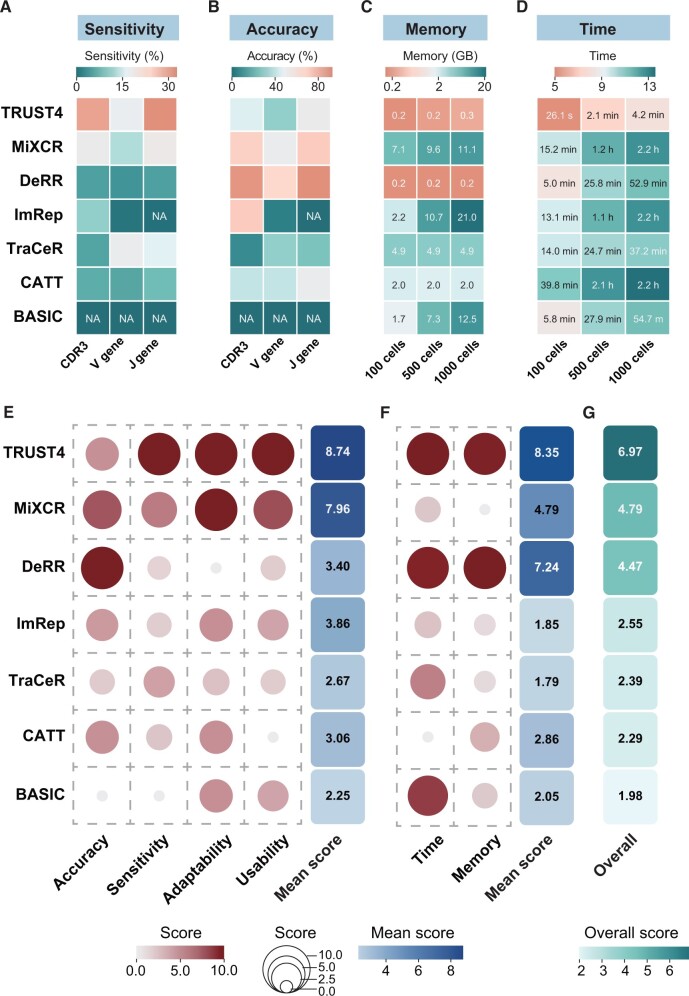
Comprehensive scoring and ranking for evaluating method performance **A**. Heatmap showing the comparison of the sensitivity of TCR reconstruction performed by different methods. **B**. Heatmap showing the comparison of the accuracy of TCR reconstruction performed by different methods. **C**. Heatmap showing the comparison of the memory usage of TCR reconstruction performed by different methods. **D**. Heatmap showing the comparison of the time consumption of TCR reconstruction performed by different methods. **E**. Left: dot plot showing the comprehensive evaluation of each method’s performance, measured using a min-max scale, where a higher score indicates better performance across accuracy, sensitivity, adaptability, and usability. Right: heatmap showing the mean scores. **F**. Left: dot plot showing the comprehensive assessment of each method’s computational consumption, also using a min-max scale, with higher scores signifying better performance in terms of time and memory efficiency. Right: heatmap showing the mean scores. **G**. Heatmap showing the overall score and ranking of each method (presented in descending order). A weight of 0.2 was assigned to sensitivity and accuracy, while a weight of 0.1 was assigned to the other factors considered. NA, not available; GB, gigabyte.

To comprehensively assess the performance, we followed established guidelines and evaluated six key aspects: accuracy, sensitivity, adaptability, usability, time consumption, and memory usage [41]. A quantitative composite score was then computed for each evaluated method (see Materials and methods). TRUST4 displayed the highest overall sensitivity, while DeRR exhibited the highest average accuracy across all tested experimental scRNA-seq data (**Figure 6**A and B). Regarding memory and time consumption, TRUST4 and DeRR exhibited more favorable performance in memory, with TRUST4 displaying the best performance in time consumption (Figure 6C and D). Additionally, the memory and time consumption of these methods fall within the acceptable limits for most computational resources. By assigning appropriate weights (0.2 for accuracy and sensitivity, and 0.1 for other aspects), we calculated an overall score for each method, subsequently ranking them accordingly (see Materials and methods). TRUST4 achieved the highest score (6.97), followed by MiXCR (4.79), DeRR (4.47), ImRep (2.55), TraCeR (2.39), CATT (2.29), and BASIC (1.98) in descending order (Figure 6E–G).

## Discussion

In this study, we conducted an extensive and rigorous benchmark analysis to evaluate the performance of seven TCR construction methods using (sc)RNA-seq datasets. We opted to exclude VDJPuzzle [26] from our evaluation due to its limited support for input data (only paired-end) and its excessive time consumption. Leveraging experimental single-cell immune profiling datasets, pseudo-bulk RNA-seq, bulk TCR-seq, and simulated scTCR-seq datasets, we systematically assessed the accuracy and sensitivity of the TCR construction. This encompassed diverse factors such as sequencing depths, read lengths, library construction strategies, and input data types. We also assessed the computational performance of each method. Overall, our study generated a guideline that can aid researchers in selecting TCR construction methods for their specific research needs.

Previous studies have benchmarked the performance of various methods for BCR construction from SMART-seq data [42]. However, the generation of BCR involves somatic hypermutations and isotype switching, which are not observed in TCR generation [4,41]. Thus, the conclusions of BCR construction methods may not be directly applied to TCR. Therefore, it is essential to independently benchmark the methods for TCR construction, as highlighted in the BCR benchmark study [42]. In this study, we also utilized the datasets generated by the popular 10X strategy and developed a simulator, YASIM-scTCR, which enables us to precisely analyze the accuracy and sensitivity of different methods.

In the context of 10X scRNA-seq data, our analysis revealed that TRUST4 and MiXCR exhibited the high sensitivity, followed by CATT and TraCeR, while DeRR and MiXCR demonstrated superior accuracy (Figure 2A and C). However, when considering bulk RNA-seq data, which generally offers deeper sequencing depths compared to scRNA-seq data, a broader range of methods including TRUST4, MiXCR, and CATT demonstrated favorable performance (Figure 5A–D). In a previous study, TRUST4 exhibited suboptimal performance for constructing BCRs from scRNA-seq datasets [29]. This discrepancy could be attributed to the intrinsic differences between the recombination processes of TCRs and BCRs or the usage of 10X scRNA-seq data which may be better suited for TRUST4 [29].

In this study, we placed particular emphasis on the influence of candidate reads, a critical factor affecting TCR construction. To facilitate analyses, we developed YASIM-scTCR, enabling the generation of scTCR-seq data with TCR- and non-TCR-derived reads, accommodating user-defined parameters such as sequencing depths and read lengths. For BCR data analysis, the simulator AIRRSHIP may offer more detailed insights for BCR benchmark studies [43]. Through simulations with YASIM-scTCR, we observed a clear association between performance over candidate read identification and TCR construction accuracy (Figure S5A and B). This underscores the key role of candidate reads in the TCR construction process. However, it is important to note that the definition of candidate reads varies across methods, which may introduce potential bias into our interpretation.

We have summarized the algorithm employed by each method (Table S5). It is evident that those with TCR-specific alignment algorithms (*e.g.*, TRUST4 and MiXCR) yield better performance than those who rely on general-purpose aligners (*e.g.*, CATT) or more naïve algorithms (*e.g.*, ImRep). In addition, assemblers with more advanced clustering algorithms (*e.g.*, TRUST4 and MiXCR) are more likely to yield better results, whereas those relying on V/J gene calling (*e.g.*, TraCeR, CATT, and ImRep) trend behave poorly. Even though not benchmarked in this study, we also noticed that certain methods use expectation-maximization (EM) algorithms in quantification (*e.g.*, TRUST4 and TraCeR), which should theoretically improve clonal expansion quantification accuracy for bulk TCR-seq data.

Using experimental datasets, our analysis suggested that the performance of most methods was not significantly influenced by species origin. However, a notable discrepancy in performance emerged between single- and paired-end sequencing data. Thus, we recommend using the paired-end mode (*i.e.*, 5′ library construction strategy) for more accurate TCR construction (Figure S6A–D). In contrast, sequencing depth emerged as a crucial determinant of accuracy. This emphasizes once again the critical importance of sequencing depth in NGS experiments [44,45].

When assessing the performance of various methods using experimental scRNA-seq data, we employed the output of CellRanger from scTCR-seq as our ground truth. scTCR-seq involves specific amplification of TCR sequences, making it a reliable source for ground truth data. While our results indicated that the performance of MiXCR, TRUST4, and DeRR was largely consistent with CellRanger on experimental scTCR-seq data, it is important to note that CellRanger itself is a method for single-cell TCR construction, which may introduce potential bias. In addition, simulated data with YASIM-scTCR provide an accurate ground truth without such potential bias.

In summary, we conducted a comprehensive evaluation of seven distinct methods across six aspects (Figure 6A–G). Among these methods, TRUST4, MiXCR, and DeRR emerged as the top performers, with TRUST4 demonstrating superior sensitivity, DeRR excelling in accuracy, and MiXCR performing well in both (Figure 6A–G). Furthermore, both TRUST4 and DeRR exhibited commendable efficiency concerning time and memory consumption. The outcome of our evaluation thus furnishes users with valuable recommendations for selecting appropriate methods tailored to their specific needs while guiding developers with valuable insights to enhance and optimize the performance of their methods.

## Materials and methods

### Datasets and preprocessing

Fourteen paired 10X scRNA-seq and scTCR-seq datasets comprising both paired- and single-end reads from human and mouse were collected in this study (Table S2). The scTCR-seq data were analyzed using CellRanger (version 6.1.2) by “cellranger vdj” command as the ground truth for each cell, while the alignment files in BAM format of scRNA-seq data were obtained by “cellranger count” command. Given that only TRUST4 and MiXCR support the 10X format while other methods do not, we split the input data into individual cells to standardize the input, ensuring compatibility across all methods for evaluation. We split the data in both FASTQ and BAM formats using a Python script, which is available on GitHub. Furthermore, we collected a total of 14 bulk TCR-seq datasets from both human and mouse and 50 SMART-seq datasets from mouse, with details provided in Table S2. All datasets can be accessed at National Center for Biotechnology Information (NCBI) (GEO: GSE114727, GSE144469, GSE160053, GSE194166, GSE223797, GSE225183, and GSE74923; BioProject: PRJNA393498 and PRJNA412649).

### TCR construction using various methods

All tools were installed on Ubuntu 20.04.4 following the installation instructions and documentation provided by each method. We referred to the methods’ user manual to determine the appropriate parameters for each run, with specific method versions and parameter settings used detailed in Table S3.

### The definition of accuracy and sensitivity

For each experimental sample, “all cells” are cells inside the “filtered_contig_annotations” output from CellRanger, whose TCRs are both full-length and productive. To evaluate the performance of each method, cells without assembly results are considered “no-result cells”, and the rest are considered “result cells”. Among the result cells, if at least one of the TCRs is correct, the cell is considered to be correctly assembled (named “true cells”). For each sample, sensitivity is defined as the percentage of true cells out of all cells in the sample, while accuracy is defined as the percentage of true cells out of the result cells. Evaluation of the V/J gene is on the subgroup level with CDR3 sequences on the amino acid level. The AsTCRs are defined as those having both the V/J genes and CDR3 sequences correct.

### The definition of candidate reads

In Figure S5, we used simulated data to explore the results of candidate read analysis among the four methods: DeRR, ImRep, TraCeR, and TRUST4. For DeRR, we modified the “DeRR.py” file by removing the line 217 “os.system(f"rm -f {sam_file} &")”. This change allowed it to keep TRJ.sam and TRV.sam, which were considered candidate reads in this study. For ImRep, we defined candidate reads as the reads mentioned in the files starting with “partial_cdr3” in its output. Regarding TraCeR, we considered candidate reads as the reads involved in TCR_A.fastq and TCR_B.fastq, located in the “aligned_reads” folder generated by the method. As for TRUST4, candidate reads were defined as the reads inside the “toassemble.fq” file.

### The definition of TCR contig depths from experimental scRNA-seq data

The calculation process for TCR contig depths is as follows: (1) identify cells with successful TCR assembly from TRUST4; (2) determine the TCR contig length; (3) calculate the candidate reads from TRUST4 output in cells where TCRs have been successfully assembled; and (4) compute the total number of nucleotides in candidate reads and divide it by the TCR contig length.

### Simulation of scTCR-seq and scRNA-seq data

We employed YASIM-scTCR (version 1.0) to simulate 10X Single Cell Immune Profiling 5′ scTCR-Seq with TCR- and non-TCR-derived reads. YASIM-scTCR is a Python tool capable of generating realistic TCR recombination events by leveraging data from huARdb [14,35]. It is available on PYPI (https://pypi.org/project/yasim-sctcr/1.0.0/), and its source code can be accessed on GitHub (https://github.com/WanluLiuLab/yasim-sctcr).

To perform the simulations, we utilized specific reference data sources for the genome and gene annotations. The soft-masked GRCh38.p12 genome sequence was obtained in FASTA format from Ensembl (release 97) and served as the reference genome for our simulations. The corresponding cDNA and peptide sequences in FASTA format of this release were also downloaded. Genes defined on chromosomes 7 and 14 with TCR-related biotypes were extracted using “seqkit grep” (version 2.5.1) [46]. The YASIM-scTCR “generate_tcr_cache” module will then prepare the references by aligning selected cDNAs and peptides using Smith-Waterman algorithm [47], which serves as the basis for generating realistic TCR recombination events during the simulations.

The process of simulating TCR rearrangements is conducted using the “rearrange_tcr” module of YASIM-scTCR. Before the generation of each TCR, whether the TCR sequence would be producible is based on a fixed probability. Firstly, TRAV, TRAJ, TRBV, and TRBJ genes are selected from the V/J gene library using the V/J usage bias pattern from huARdb (Figure S4). Then, the V/J gene clipping on the TRA and TRB chains is performed based on statistics from huARdb (Figure S3A and B). Subsequently, CDR3 sequences are generated according to the statistics from huARdb (Figure S3C). Furthermore, V/J genes are subjected to additional clipping criteria to ensure that the V gene starts with cysteine (C) and the J gene ends with phenylalanine (F, for most genes) or tryptophan (W, for TRAJ33, TRAJ38, and TRAJ55 only). In cases where the generation fails, the simulator retries until successful, ensuring the production of reliable and realistic TCR sequences. Approximately 50 bp of the corresponding C gene segment is then added, mimicking the behavior of C-specific primers in scTCR-seq. The generated ground-truth TCR contigs are exported in FASTA format, preserving the ground-truth V/J gene names and CDR3 sequences.

Even though not considered in our simulated data, YASIM-scTCR also supports simulation for TCR clone expansion level when generating a large amount of scTCR-seq data. After the introduction of TCR clonal expansion (Zipf’s distribution) and sequencing depth (uniform distribution), the mRNA contigs are reverse-complemented to allow 5′ amplification using seqtk (https://github.com/lh3/seqtk). Finally, driven by YASIM-scTCR, ART [36] with the “--amplicon” parameter is utilized with desired sequencing length and machine errors, providing simulated 5′ scTCR-seq data containing TCR-derived reads.

The simulation of scTCR-seq data with non-TCR-derived reads involves several steps. Firstly, the reference mRNAs are filtered for protein-coding genes (with “protein_coding” biotype) selected by Matched Annotation from NCBI and EMBL-EBI (MANE) [48]. For expression data, we used scDesign2 [36] trained by the HU_0043_Blood_10X dataset from the Human Universal Single Cell Hub (HUSCH) database on HUGO Gene Nomenclature Committee (HGNC)-approved MANE-selected protein-coding genes [49,50]. YASIM-scTCR could also accept count matrices in other formats that could be parsed by AnnData [51]. The expression data are manually scaled to a mean depth of 1× for each gene per cell. All transcripts are reverse-complemented and passed to YASIM-scTCR. Additionally, each cell is barcoded to facilitate downstream analysis and identification.

### Pseudo-bulk RNA-seq data for TCR construction analysis

We randomly selected 100, 500, and 1000 individual cells from the scRNA-seq data of FASTQ and BAM formats. These selected cells were combined into pseudo-bulk RNA-seq data. BAM files were merged using SAMtools (version 1.15.1), while the FASTQ files were concatenated using the command “cat”. During the evaluation process, we recorded the time and memory consumption for each method using GNU Time.

### Scoring principles and standards

The evaluation scores presented in Figure 6E are derived from six attributes, as detailed in Table S4.

For the accuracy score, the proportion of cells correctly assembled for CDR3 and V/J gene in each of the 14 samples was calculated and averaged, with weights of 0.5, 0.3, and 0.2 respectively. To transform the accuracy score into a more interpretable range, it was scaled to a 1–10 scale using the min-max scaling method. Sensitivity scores were calculated in a similar way.

The time and memory scores for each method were calculated using log_2_ [1/time (s)] and log_2_ [1/memory (kB)] with varying cell numbers, and scaled to 1–10. Higher scores indicate better time and memory performance. The details of the time, memory, and CPU usage are available in Table S4.

The adaptability score was determined by evaluating the number of input formats supported by each method, considering factors such as cell type (T/B cell), input data format (FASTQ/BAM files), and species supported (human or mouse). One point was awarded for each aspect, and the overall adaptability score was scaled to a 1–10 range.

Usability evaluation focused on two aspects: the quality and variety of the method’s output files and the user-friendliness of the method. Four output files (candidate reads, assembled reads, contig sequences, and annotation contigs) were considered, with one point awarded for each file supported. Additionally, the user-friendliness score, based on subjective evaluation, was assigned a maximum of 5 points. They were combined to obtain the overall usability score, which was then scaled to a 1–10 range.

The min-max scale formula is shown below:


$$x=\alpha +\frac{\left(X-X_{\mathrm{Min}})(b-a\right)}{X_{\mathrm{Max}}-X_{\mathrm{Min}}}.$$

##  

where *X* is the original value of the feature to be scaled, *X*_Min_ is the minimum value of the feature in the dataset, *X*_Max_ is the maximum value of the feature in the dataset, *a* is the lower bound of the desired scale range, and *b* is the upper bound of the desired scale range.

## Code availability

The source code of YASIM-scTCR can be accessed for research purposes at BioCode (https://ngdc.cncb.ac.cn/biocode/tool/BT7591), Github (https://github.com/WanluLiuLab/yasim-sctcr), and PYPI (https://pypi.org/project/yasim-sctcr/1.0.0/). Documentation of YASIM-scTCR can be found at https://labw.org/yasim-sctcr-docs/. The code for statistics and visualization in this study can be accessed at GitHub (https://github.com/WanluLiuLab/Benchmarking_TCR_Construction).

## CRediT author statement

**Ruonan Tian:** Conceptualization, Methodology, Software, Formal analysis, Visualization, Writing – original draft. **Zhejian Yu:** Conceptualization, Methodology, Software, Writing – original draft. **Ziwei Xue:** Data curation, Writing – review & editing. **Jiaxin Wu:** Software. **Lize Wu:** Writing – review & editing. **Shuo Cai:** Visualization. **Bing Gao:** Data curation. **Bing He:** Funding acquisition. **Yu Zhao:** Funding acquisition. **Jianhua Yao:** Funding acquisition. **Linrong Lu:** Funding acquisition. **Wanlu Liu:** Conceptualization, Supervision, Project administration, Funding acquisition, Writing – review & editing. All authors have read and approved the final manuscript.

## Supplementary material

Supplementary material is available at *Genomics, Proteomics & Bioinformatics* online (https://doi.org/10.1093/gpbjnl/qzae086).

## Competing interests

Bing He, Yu Zhao, and Jianhua Yao are current employees of Tencent Technology (Shenzhen) Co., Ltd. All the other authors have declared no competing interests.

## Supplementary Material

qzae086_Supplementary_Data
