# Supplementary material for: Evaluation of T Cell Receptor Construction Methods from scRNA-Seq Data
Source: Genomics Proteomics Bioinformatics. 2024 Dec 12;22(6):qzae086. doi: 10.1093/gpbjnl/qzae086 (PMC11846667; doi:10.1093/gpbjnl/qzae086)
Supplement: qzae086_Supplementary_Data [file qzae086_supplementary_data.zip › supplementary material captions.docx]

**Supplementary material**

**Figure S1 Basic statistics related to TCR**

**A.** Box plot represents the UMI counts of human CDR3α and CDR3β sourced from huARdb, accompanied by a paired Wilcoxon signed-rank test yielding a *P* value of 2.2E–16.

**Figure S2 Comparative analysis of TCR construction methods on SMART-seq data**

**A.** Histogram represents the number of cells for each method successfully reconstructed the AsTCR. **B.** Upset plots represent the overlap of unique CDR3α (left) and CDR3β (right) sequences assembled by each method. **C.** Upset plots represent the overlap of unique J gene α (left) and J gene β (right) sequences assembled by each method. **D.** Upset plots represent the overlap of unique V gene α (left) and V gene β (right) sequences assembled by each method.

**Figure S3 TCR CDR3 insertion and V/J gene deletion amino acid statistics**

**A.** Density plot represents the amino acid length distribution of assembled human TCR sourced from huARdb. **B.** Histogram represents the amino acid length distribution of human CDR3α sourced from huARdb. **C.** Histogram represents the amino acid length distribution of human CDR3β sourced from huARdb. **D.** Histogram represents the distribution of deleted amino acids number in the junction with CDR3 of V/J genes. **E.** Histogram represents the distribution of amino acid lengths of J gene. **F.** Logo plot of amino acid preference statistics for CDR3 of TCRα with different amino acid lengths. Each letter in the logo plot corresponds to a specific amino acid, represented by its single-letter code and the height of the letters in the logo plot indicates the relative frequency of each amino acid at that position. **G.** Logo plot of amino acid preference statistics for CDR3 of TCRβ with different amino acid lengths.

**Figure S4 The usage of V/J gene in experimental data**

**A.** Sankey plot represents the usage of V/J gene in experimental data. Each node represents each gene. Only V/J genes with a frequency greater than 0.05 were retained. Left, TRAV gene; Right, TRAJ gene. **B.** Sankey plot represents the usage of V/J gene in experimental data. Each node represents each gene. Only V/J genes with a frequency greater than 0.05 were retained. Left, TRBV gene; Right, TRBJ gene.

**Figure S5 The statistics of candidate reads obtained from simulated scTCR-seq datasets**

**A.** Confusion matrix for finding candidate reads in simulated scTCR-seq and scRNA-seq datasets (reads length: 250 base pair, reads depth: 400**×**). TP, true positive; FP, false positive; FN, false negative; TN, true negative. **B.** Bar plot of four methods in terms of the accuracy of simulated datasets (reads length: 250 base pair, reads depth: 400**×**).

**Figure S6 Performance comparison of methods on multiple data types**

**A.** Dot plot represents the sensitivity of different methods for mouse and human data. **B.** Dot plot represents the accuracy of different methods for mouse and human data. **C.** Dot plot represents the sensitivity of different methods for paired-end (PE) and single-end (SE) data. **D.** Dot plot represents the accuracy of different methods for paired-end (PE) and single-end (SE) data.

**Figure S7 Performance comparison on simulated data with different read lengths**

**A.** Bar plot represents the accuracy of TCR assembled by different method under varying read lengths. **B.** Bar plot represents the sensitivity of TCR assembled by different method under varying read lengths.

**Figure S8 Performance comparison on simulated data with different sequencing depths**

**A.** Box plot represents the total read number of CDR3, J gene and V gene in cells with successfully (True) and unsuccessfully (False) assembled TCR on the collected real scRNA-seq datasets. **B.** Bar plots represent the method accuracy in assembling TCRα in simulated data with different sequencing depth. **C.** Bar plots represent the method accuracy in assembling TCRβ in simulated data with different sequencing depth.

**Figure S9 Comparative analysis of CDR3β construction methods on bulk TCR-seq data**

**A.** Box plot represents the number of unique CDR3β sequences assembled by each method. **B.** Upset plots represent the overlap of unique CDR3β sequences assembled by each method.

**Figure S10 Performance assessment of methods on pseudo bulk RNA-seq with varying TCR abundance**

**A.** Box plot represent the sensitivity of TCRα assembled by different methods at varying TCR abundance. The line in the middle represents the median; boxes represent the 25th (bottom) and 75th (top) percentiles; and whiskers represent the minimum and maximum points within 1.5 times the interquartile range. **B.** Box plot represent the sensitivity of TCRβ assembled by different methods at varying TCR abundance. **C.** Box plot represent the accuracy of TCRα assembled by different methods at varying TCR abundance. **D.** Box plot represent the accuracy of TCRβ assembled by different methods at varying TCR abundance.

**Table S1** **Summary of tools**

**Table S2** **Summary of experimental datasets**

**Table S3** **The version and code of tools**

**Table S4** **The details of each tool’s performance**

**Table S5** **Description of algorithm employed by different tools**
