# Supplementary figures and images for: Evaluation of T Cell Receptor Construction Methods from scRNA-Seq Data
Source: Genomics Proteomics Bioinformatics. 2024 Dec 12;22(6):qzae086. doi: 10.1093/gpbjnl/qzae086 (PMC11846667; doi:10.1093/gpbjnl/qzae086)

**A**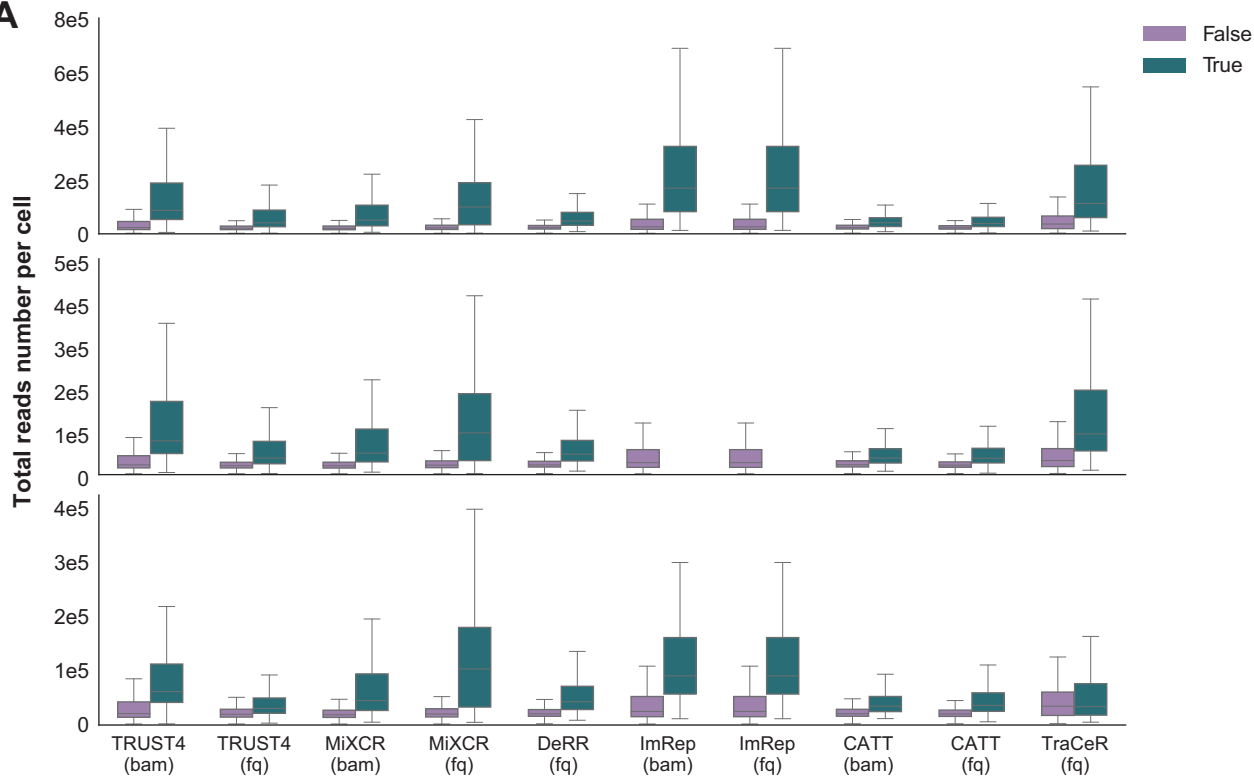**B****TCR $\alpha$** 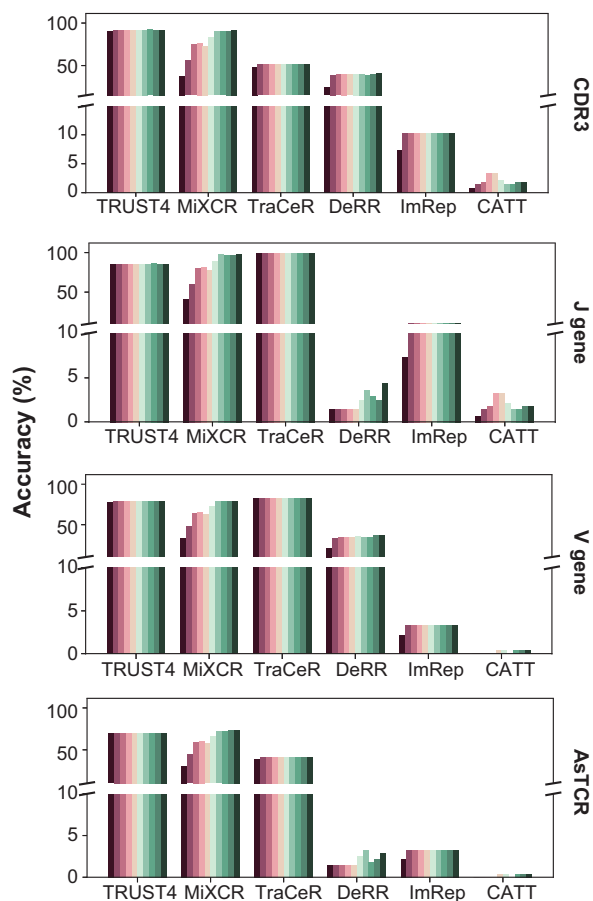**C****TCR $\beta$** 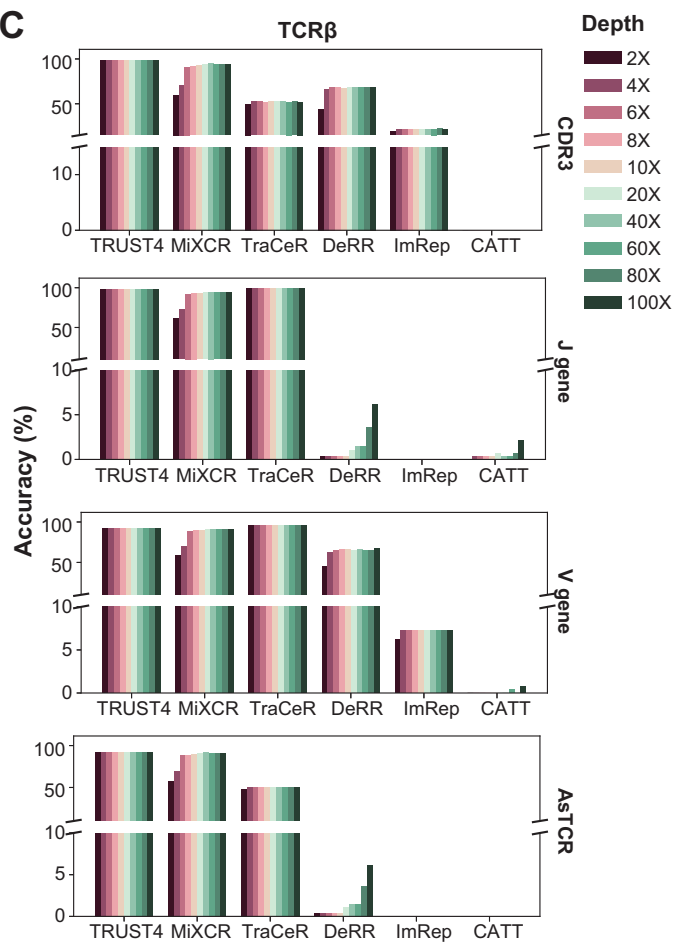

Supplement: qzae086_Supplementary_Data [file qzae086_supplementary_data.zip › Figure S8.pdf]

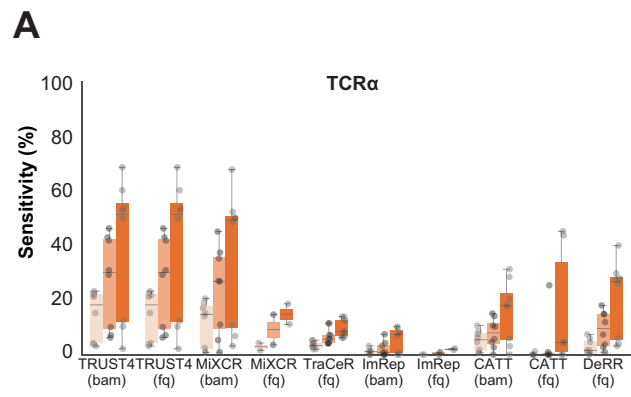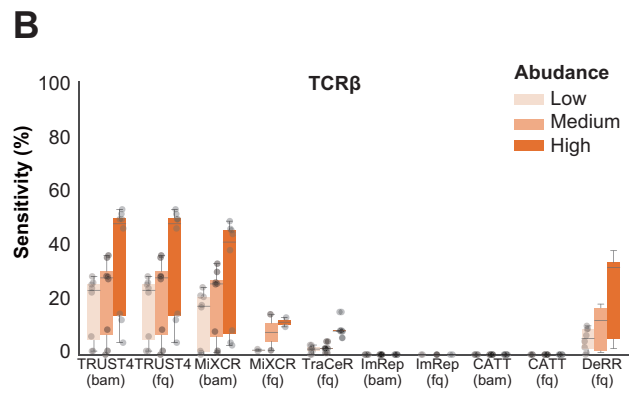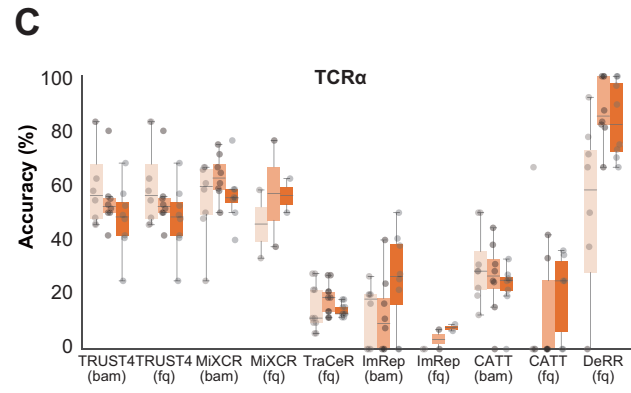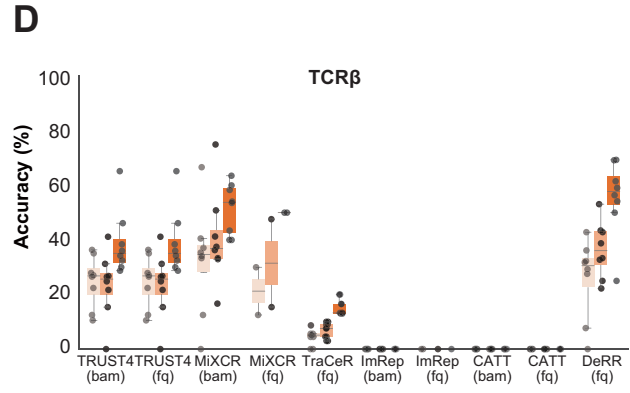

Supplement: qzae086_Supplementary_Data [file qzae086_supplementary_data.zip › Figure S10.pdf]

**A**

100bp 150bp 250bp

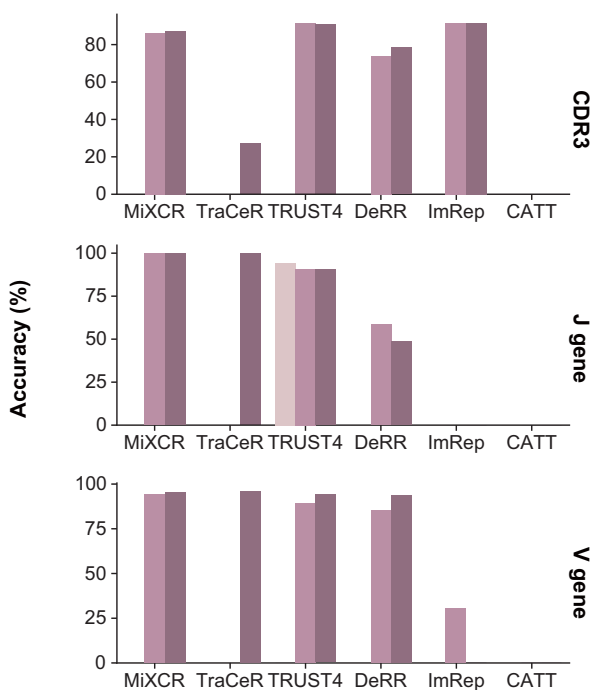**B**

100bp 150bp 250bp

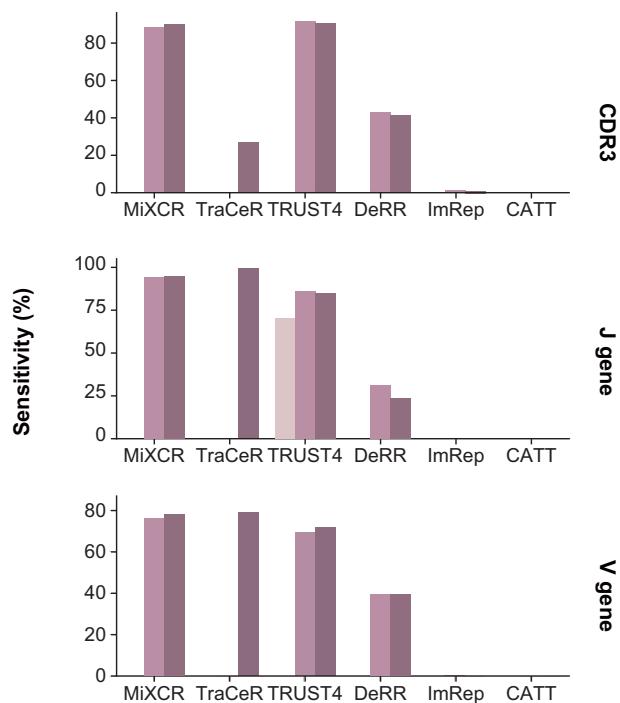

Supplement: qzae086_Supplementary_Data [file qzae086_supplementary_data.zip › Figure S7.pdf]

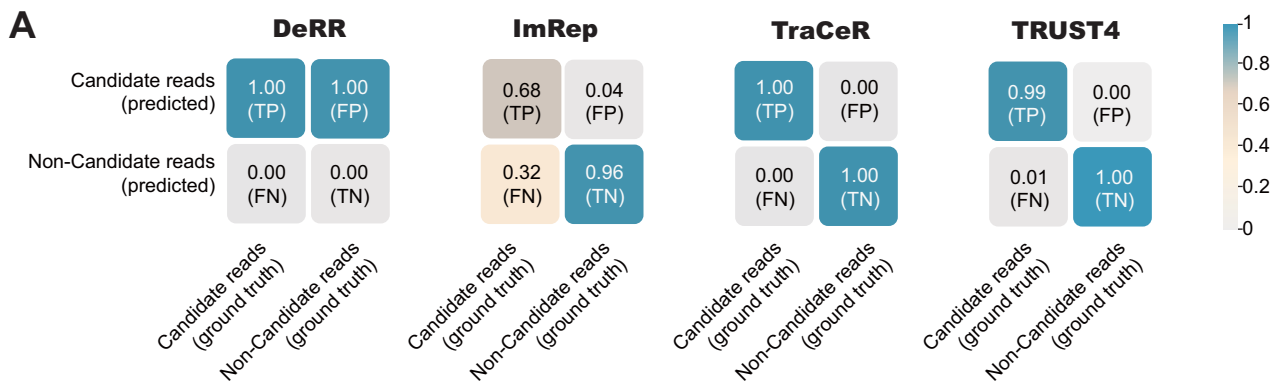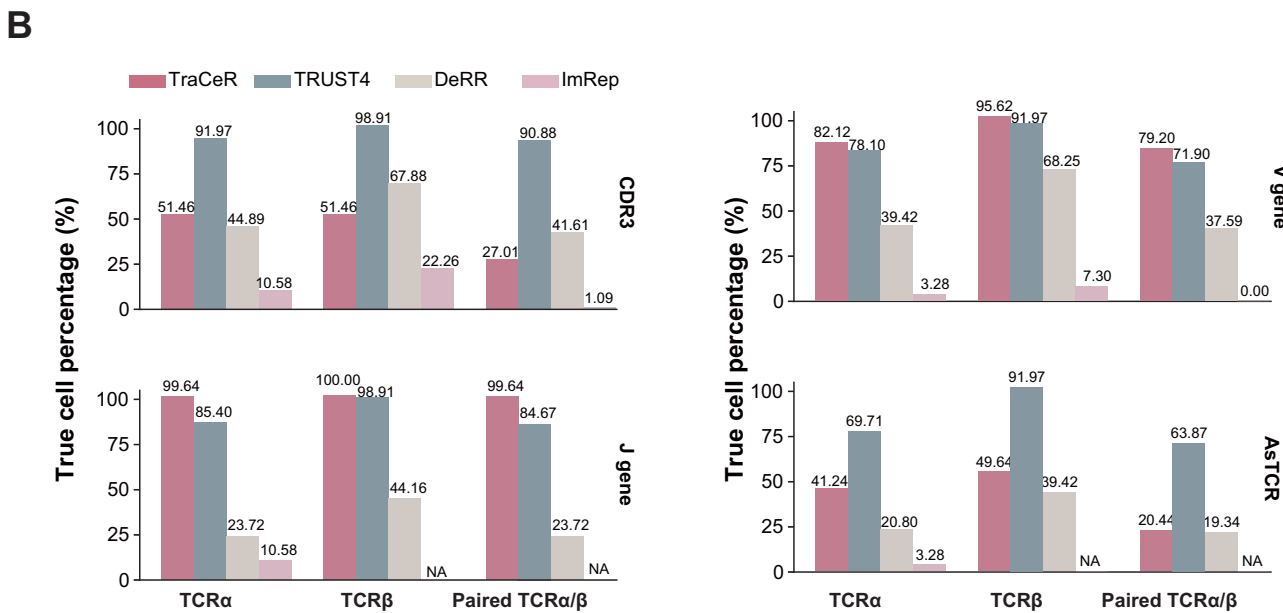

Supplement: qzae086_Supplementary_Data [file qzae086_supplementary_data.zip › Figure S5.pdf]

**A**

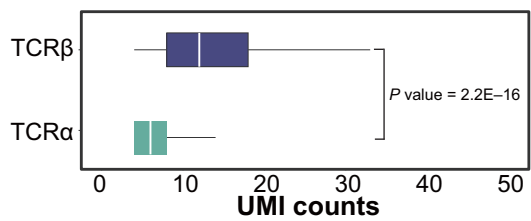

Supplement: qzae086_Supplementary_Data [file qzae086_supplementary_data.zip › Figure S1.pdf]

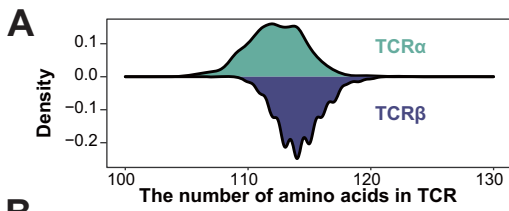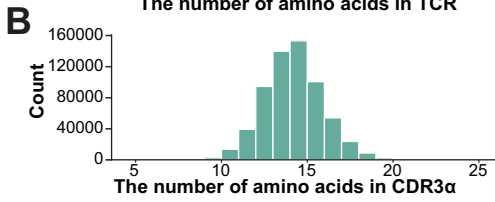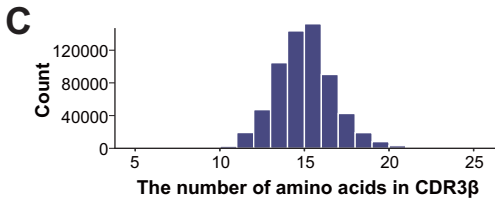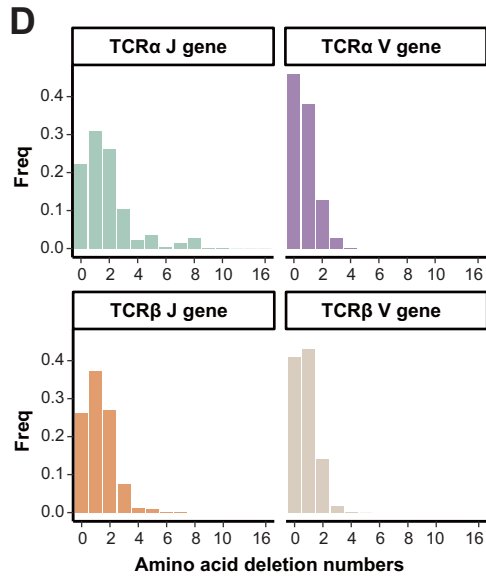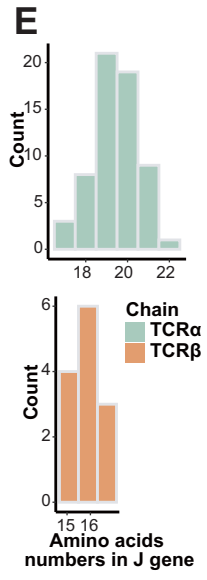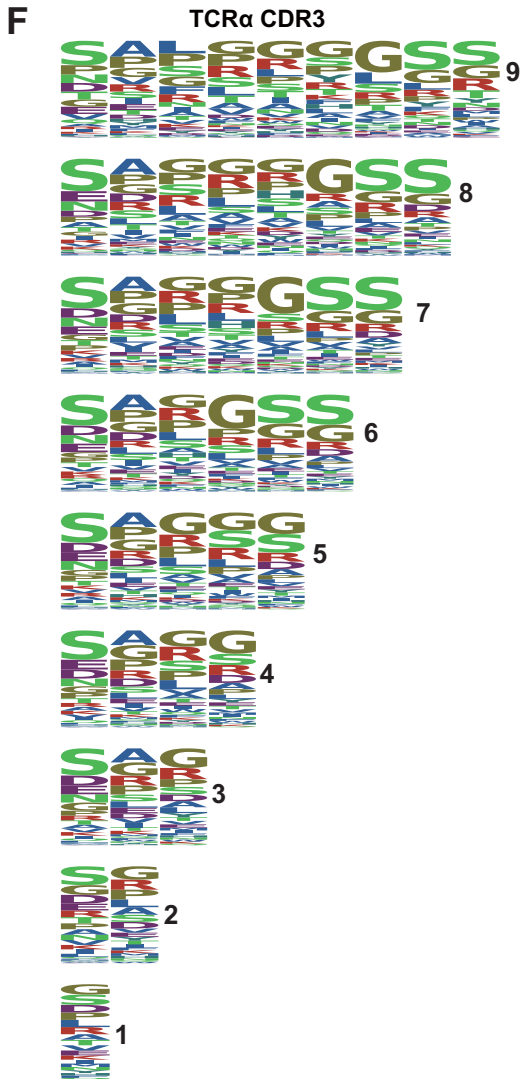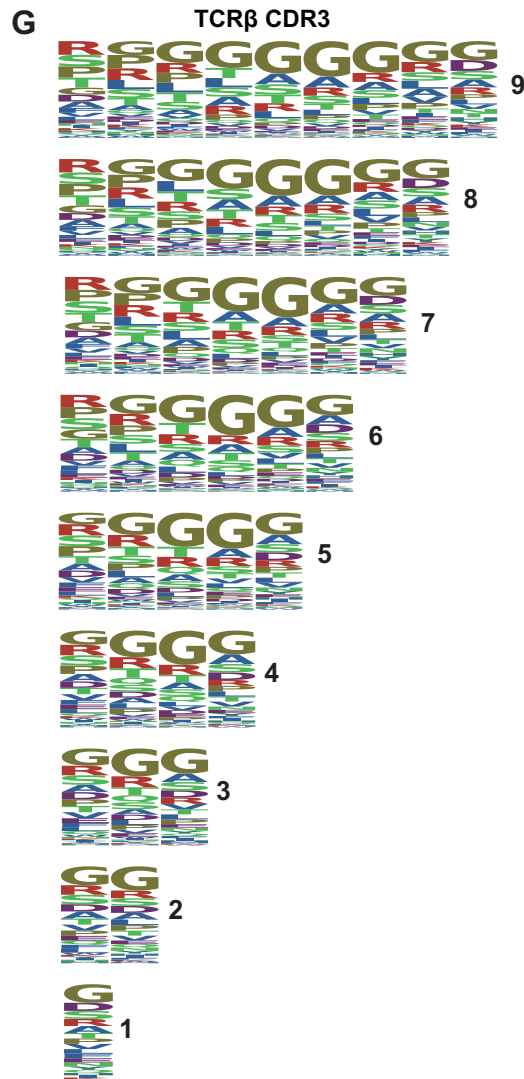

Supplement: qzae086_Supplementary_Data [file qzae086_supplementary_data.zip › Figure S3.pdf]

**A**

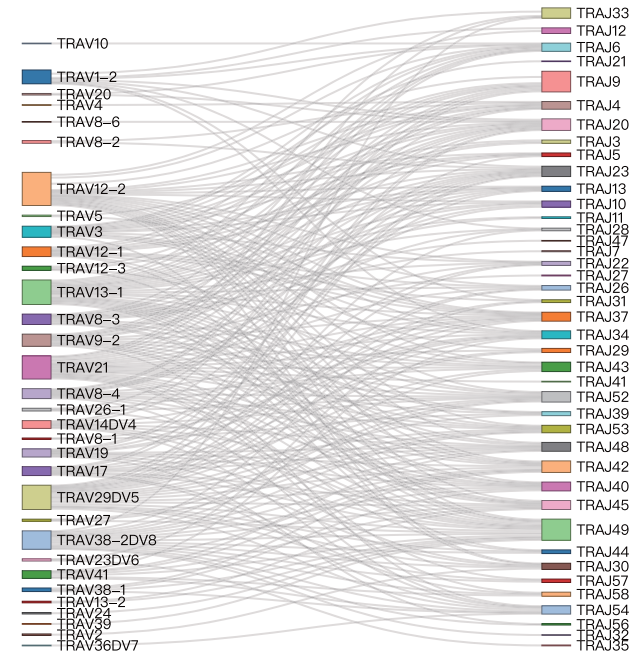

**B**

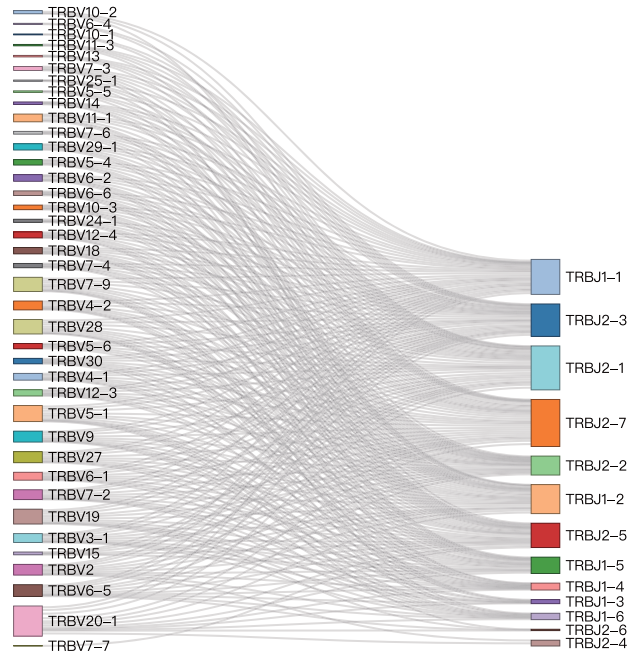

Supplement: qzae086_Supplementary_Data [file qzae086_supplementary_data.zip › Figure S4.pdf]

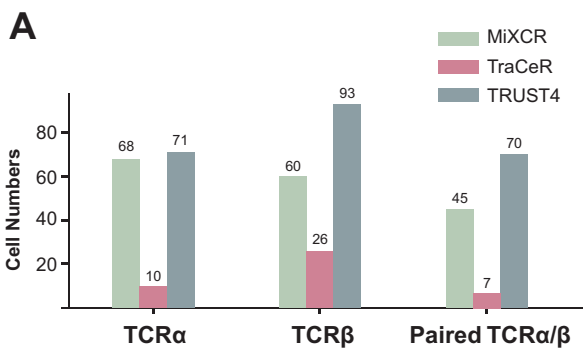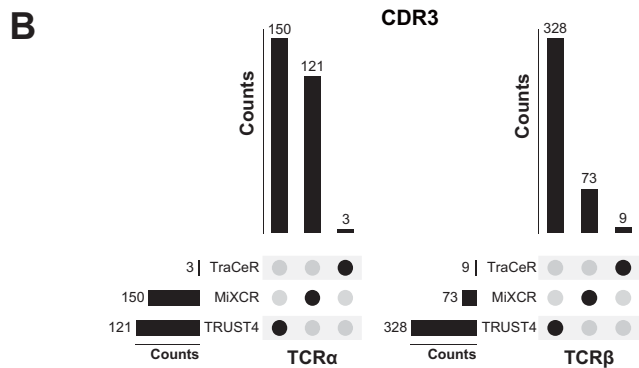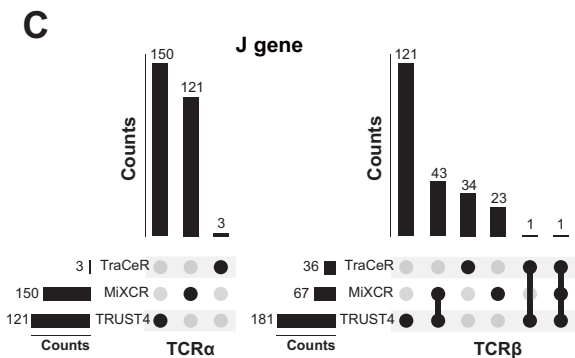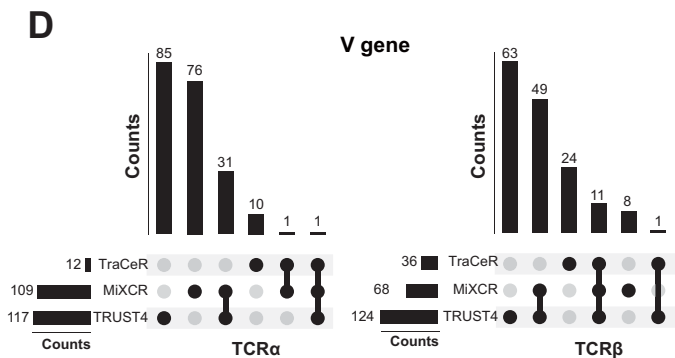

Supplement: qzae086_Supplementary_Data [file qzae086_supplementary_data.zip › Figure S2.pdf]

A

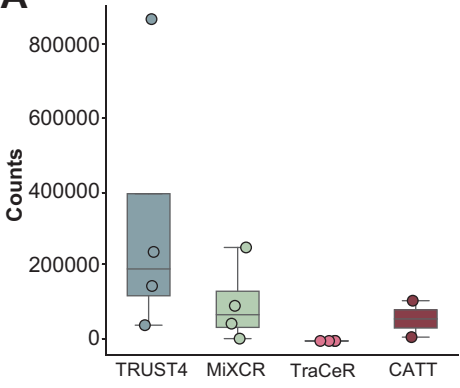

B

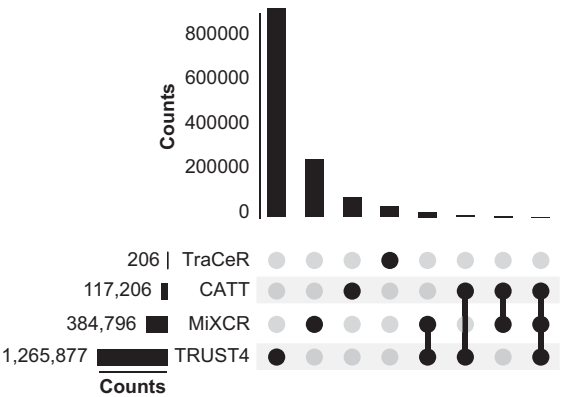

Supplement: qzae086_Supplementary_Data [file qzae086_supplementary_data.zip › Figure S9.pdf]

**A**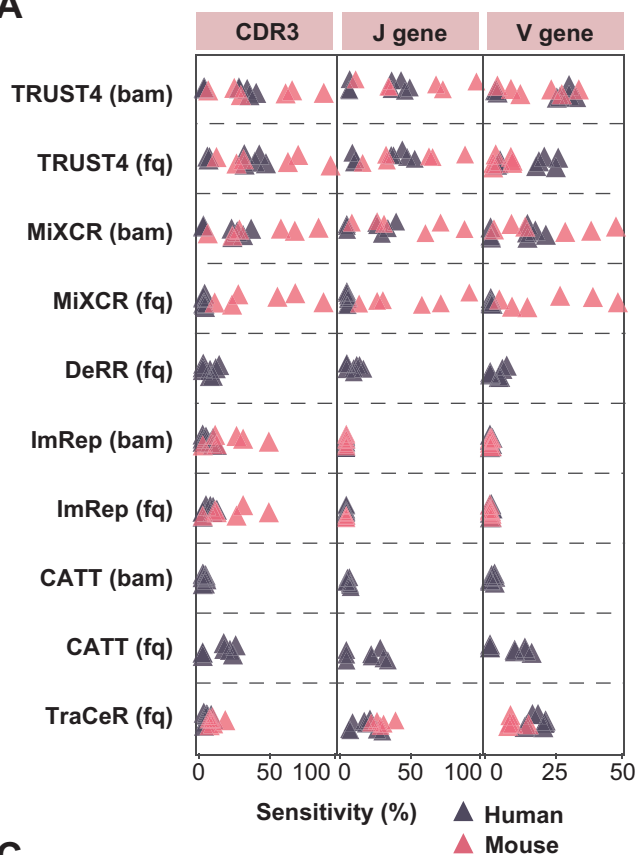**B**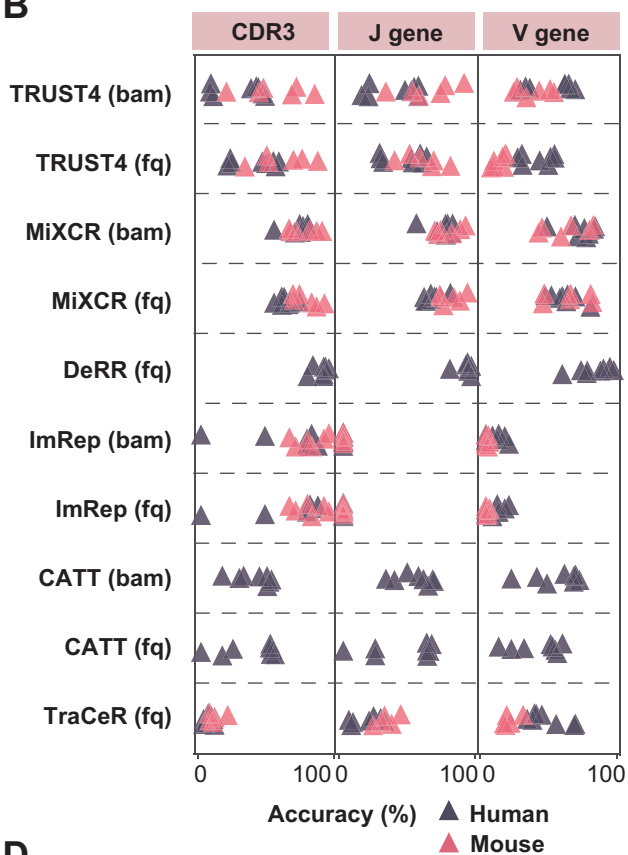**C**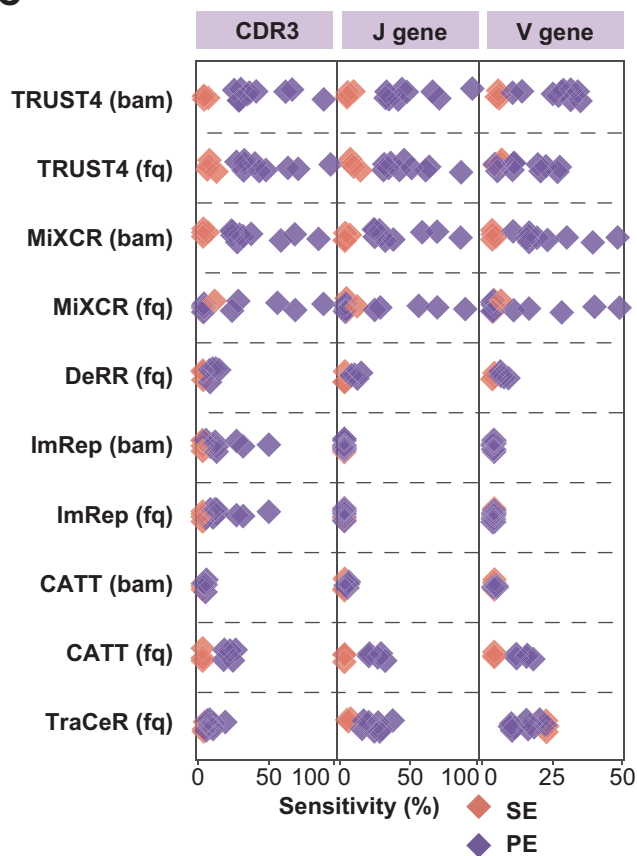**D**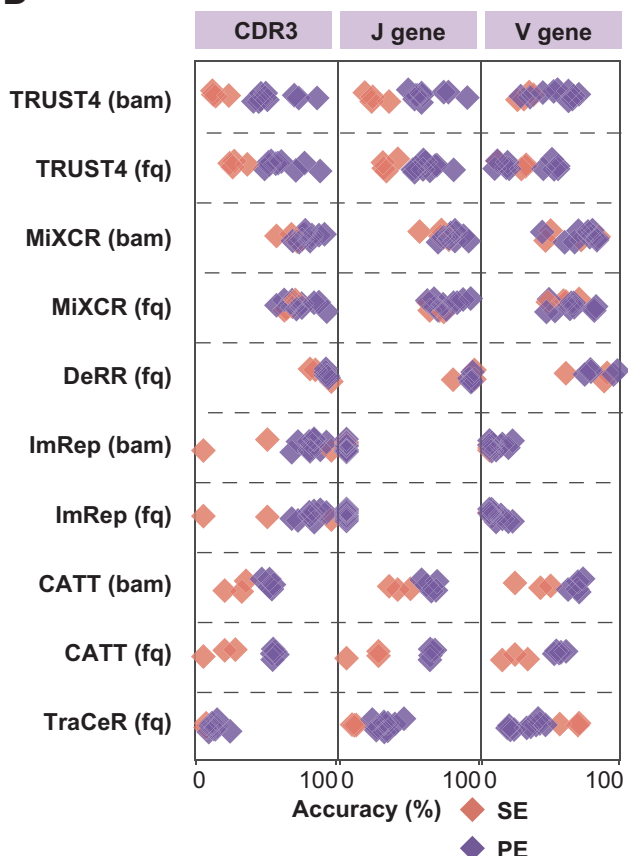

Supplement: qzae086_Supplementary_Data [file qzae086_supplementary_data.zip › Figure S6.pdf]
